# Endoplasmic reticulum stress induces ligand-independent TNFR1-mediated necroptosis in L929 cells

**DOI:** 10.1038/cddis.2014.548

**Published:** 2015-01-08

**Authors:** S Saveljeva, S L Mc Laughlin, P Vandenabeele, A Samali, M J M Bertrand

**Affiliations:** 1 Apoptosis Research Center, National University of Ireland, Galway, Ireland; 2 School of Natural Sciences, National University of Ireland, Galway, Ireland; 3 VIB Inflammation Research Center (IRC), Technologiepark 927, Zwijnaarde-Gent 9052, Belgium; 4 Department of Biomedical Molecular Biology, VIB/Ghent University, Technologiepark 927, Zwijnaarde-Gent 9052, Belgium

## Abstract

Endoplasmic reticulum (ER) stress-induced cellular dysfunction and death is associated with several human diseases. It has been widely reported that ER stress kills through activation of the intrinsic mitochondrial apoptotic pathway. Here we demonstrate that ER stress can also induce necroptosis, an receptor-interacting protein kinase 1 (RIPK1)/RIPK3/mixed lineage kinase domain-like protein (MLKL)-dependent form of necrosis. Remarkably, we observed that necroptosis induced by various ER stressors in L929 cells is dependent on tumor necrosis factor receptor 1 (TNFR1), but occurs independently of autocrine TNF or lymphotoxin *α* production. Moreover, we found that repression of either TNFR1, RIPK1 or MLKL did not protect the cells from death but instead allowed a switch to ER stress-induced apoptosis. Interestingly, while caspase inhibition was sufficient to protect TNFR1- or MLKL-deficient cells from death, rescue of the RIPK1-deficient cells additionally required RIPK3 depletion, indicating a switch back to RIPK3-dependent necroptosis in caspase-inhibited conditions. The finding that ER stress also induces necroptosis may open new therapeutic opportunities for the treatment of pathologies resulting from unresolved ER stress.

The endoplasmic reticulum (ER) has a major role in the synthesis, folding and trafficking of secretory and membrane proteins.^1^ Many cellular conditions can alter proper ER functions. As a consequence, un- or misfolded proteins accumulate in the ER lumen and induce ER stress. All eukaryotic cells have developed a quality control system, known as the unfolded protein response (UPR), to sense and adapt to ER stress.^2^ In mammalian cells, the UPR emerges from three ER-anchored receptors (inositol-requiring enzyme-1 (IRE1), protein kinase RNA-like ER kinase (PERK) and activating transcription factor 6) and promotes a return to ER homeostasis by activating signaling pathways aimed at increasing the folding capacity of the ER, reducing synthesis of new proteins and promoting alternative forms of protein degradation (such as ER-associated degradation and autophagy). However, when ER stress is too severe and/or prolonged, the UPR is insufficient to restore homeostasis, and therefore turns into a toxic signal leading to cell death.^3, 4^ Accumulating evidence indicate that ER stress-induced cellular dysfunction and death are associated with and contribute to several human diseases (such as neurodegenerative diseases, inflammation and cancer), highlighting the need for a better understanding of the molecular mechanisms regulating ER stress-mediated death in the hope to identify new therapeutic targets.^5, 6, 7^

ER stress is widely reported to induce caspase-dependent apoptotic cell death, and although few studies support implication of the receptor extrinsic pathway, the vast majority of them attribute the killing to the activation of the mitochondrial intrinsic pathway.^4^ The intrinsic apoptotic pathway relies on the B-cell lymphoma 2 (BCL-2)-associated X protein/BCL-2 antagonist/killer-dependent mitochondrial outer membrane permeabilization (MOMP), which causes the release of cytochrome *c* into the cytoplasm and allows formation of the apoptosome and the subsequent activation of procaspase-9. Distinct mechanisms have been reported to induce MOMP by modulating the expression and/or activation of the various pro- and anti-death BCL-2 family members in conditions of unresolved ER stress.^8^ Among them are the IRE1-mediated c-Jun N-terminal kinase (JNK) activation,^9^ the controversial IRE1-dependent degradation of caspase-2 targeting miRNA^10, 11^ or the PERK-dependent expression of the transcription factor C/EBP-homologous protein (CHOP).^12, 13^

Apoptosis is however not the only way for a cell to die, and recent studies have highlighted the importance of necroptosis, a regulated form of necrosis that relies on the enzymatic activity of the serine/threonine receptor-interacting protein kinase 1 (RIPK1) and RIPK3, in the pathogenesis of various human diseases.^14, 15^ Necroptosis has so far mainly been studied in the context of death receptor signaling, such as downstream of the tumor necrosis factor (TNF) receptor 1 (TNFR1), and was shown to prevail in caspase-8-inhibited conditions.^16, 17, 18^ In contrast to most cells, the murine fibrosarcoma L929 cells do not require caspase inhibition to undergo TNF-mediated necroptosis, rendering these cells of particular interest for the *in vitro* study of necroptosis. Nevertheless, L929 cells retain the ability to undergo apoptosis, and switches to TNF-mediated apoptosis have been reported when components of the necroptotic machinery are repressed,^19, 20^ L929 cells also activate the intrinsic apoptotic pathway when stimulated with apoptosis-inducing agents such as staurosporine.^21^

In this study, we investigated the cell death modality induced by sustained ER stress in the L929 cell line, a cellular model for both apoptosis and necroptosis induction. In these cells, we found that ER stress induction by chemical triggers such as brefeldin A, thapsigargin and tunicamycin did not activate the intrinsic apoptotic pathway but instead triggered TNFR1-mediated necroptosis. Remarkably, TNFR1 signaling was independent of the autocrine production of the receptor's cognate ligands TNF and lymphotoxin *α* (LT*α*). In addition, we found that chemical inhibition of RIPK1 kinase activity by necrostatin-1 (Nec-1) protected the cells from death, while repressing RIPK1, mixed lineage kinase domain-like protein (MLKL) or TNFR1 levels induced a switch in the cell death modality, from necroptosis to apoptosis. Taken together, these results highlight the ability of ER stress to trigger cell death modalities other than apoptosis, and identified Nec-1 as a potential new compound for the treatment of pathologies resulting from unresolved ER stress-mediated death.

## Results

### ER stress induces caspase-independent necroptosis in L929sA cells

**Figure 1:**
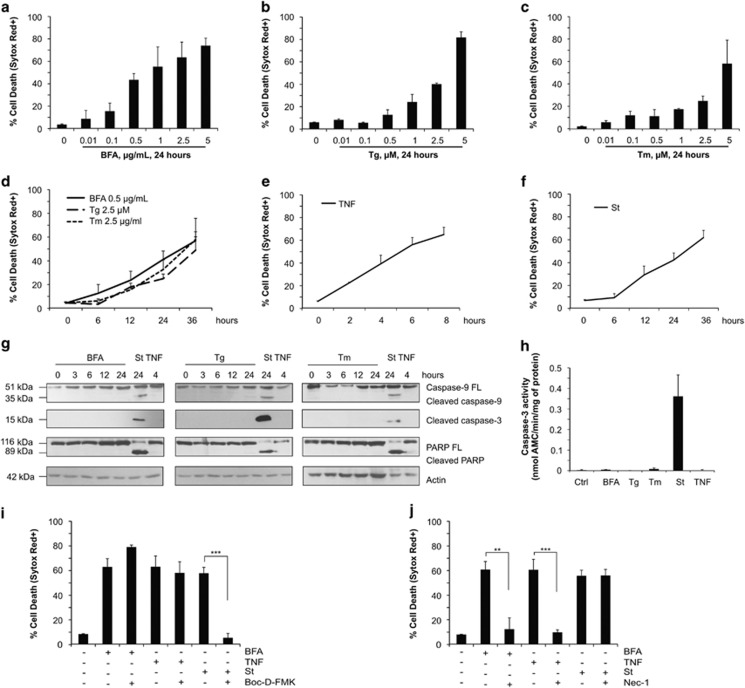
ER stress induces necroptosis in L929sA cells. (**a**–**f**) L929sA cells were treated with increasing concentrations of brefeldin A (BFA) (**a**), thapsigargin (Tg) (**b**) and tunicamycin (Tm) (**c**) for 24 h, with 0.5 *μ*g/ml of BFA, 2.5 *μ*M of Tg and 2.5 *μ*g/ml of Tm over a period of 36 h (**d**) or with 30 ng/ml of human TNF (hTNF) (**e**) up to 8 h or with 10 nm of staurosporine (St) up to 36 h (**f**), and the percentage of cell death was determined by flow cytometry following Sytox Red staining. (**g**) Immunoblots of cell lysates isolated following treatment with 0.5 *μ*g/ml of BFA, 2.5 *μ*g/ml of Tm and 2.5 *μ*M of Tg for the indicated period, as well as with 10 nM of St (24 h) and 30 ng/ml of hTNF (4 h). (**h**) Caspase-3 activity measured by DEVD-AMC assay after 24 h of treatment with 0.5 *μ*g/ml of BFA, 2.5 *μ*g/ml of Tm and 2.5 *μ*M of Tg, as well as after 24 h with 10 nM of St and 4 h with 30 ng/ml of hTNF. (**i** and **j**) Cell death evaluated by flow cytometry after Sytox Red staining of L929sA stimulated with 0.5 *μ*g/ml of BFA (24 h), 10 nM of St (24 h) and 30 ng/ml of hTNF (4 h) in the absence and presence of 10 *μ*M of Boc-D-FMK (**i**) or 20 *μ*M of Nec-1 (**j**). PARP, poly-ADP ribose polymerase

To determine the cell death modality induced by sustained ER stress in the L929sA cell line, we stimulated the cells with three different compounds reported to be strong inducers of ER stress, namely brefeldin A, thapsigargin and tunicamycin. ER stress induction by these compounds was confirmed by monitoring the expression of several ER stress markers, such as spliced XBP-1, Grp78 and CHOP (Supplementary Figure S1). We observed that each compound triggered death of the L929sA cells in a dose-dependent manner (Figures 1a–c), and that ~30–50% of cell death was obtained after 24 h of stimulation with the different compounds used at the respective concentration of 0.5 *μ*g/ml, 2.5 *μ*M and 2.5 *μ*g/ml (Figure 1d). A comparable amount of cell death was obtained by stimulating the cells with either 30 ng/ml of TNF for 4 h or with 10 nM of staurosporine for 24 h, two triggers, respectively, used as positive controls for necroptosis and apoptosis induction in these cells (Figures 1e and f). To evaluate whether the death induced by the different ER stressors was relying on caspase activation, we monitored processing of caspase-9 and -3 as well as cleavage of the caspase-3 substrate PARP by immunoblotting cell lysates obtained after stimulating the cells with 0.5 *μ*g/ml of brefeldin A, 2.5 *μ*M of thapsigargin and 2.5 *μ*g/ml of tunicamycin over a period of 24 h. Similarly to TNF stimulation and contrary to staurosporine treatment, none of the ER stress inducers lead to caspase activation (Figure 1g), which was confirmed by DEVDase assays performed on lysates collected 24 h after stimulation (Figure 1h). In line with these results, pre-treatment with the pancaspase inhibitor Boc-D-FMK protected the cells from staurosporine-mediated killing but not from brefeldin A- or TNF-induced death (Figure 1i). To test whether the caspase-independent death caused by the administration of the ER stressors could result from necroptosis induction, we next evaluated the potential protective effect of inhibiting RIPK1 kinase activity with Nec-1. As shown in Figure 1j, RIPK1 kinase inhibition protected L929sA cells from brefeldin A-induced death as efficiently as following TNF stimulation, but had no effect on staurosporine-mediated killing. We confirmed these results using the more stable and specific RIPK1 inhibitor, Nec-1s (Supplementary Figure S2). Taken together, these results demonstrate that ER stress induces RIPK1 kinase-dependent necroptosis in L929 cells.

### RIPK1 repression shifts ER stress-induced necroptosis to ER stress-induced apoptosis

**Figure 2:**
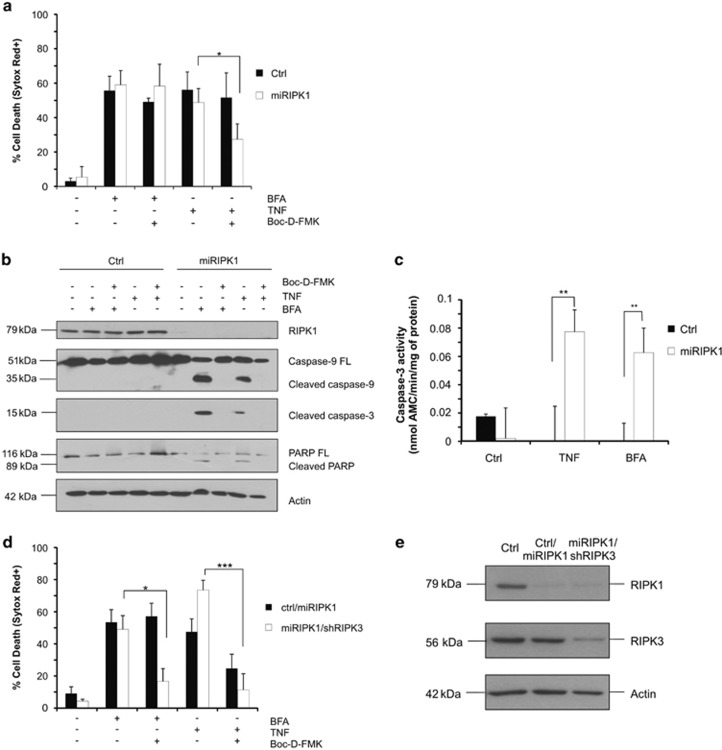
RIP1K1 repression shifts ER stress-induced necroptosis to ER stress-induced apoptosis. (**a**) Control (Ctrl) and RIPK1-depleted (miRIPK1) L929sA cells were treated for 24 h with 0.5 *μ*g/ml of brefeldin A (BFA) or 2–4 h with 30 ng/ml of human TNF (hTNF) (Ctrl cells were stimulated for 4 h and RIPK1-depleted cells for 2 h to induce a comparable amount of cell death), alone or in combination with 10 *μ*M of Boc-D-FMK, and viability was evaluated by flow cytometry following Sytox Red staining. (**b**) Immunoblots of cell lysates of Ctrl and RIPK1-depleted L929sA cells isolated following treatment for 24 h with 0.5 *μ*g/ml of BFA or 2–4 h with 30 ng/ml of hTNF (Ctrl cells were stimulated for 4 h and RIPK1-depleted cells for 2 h to induce a comparable amount of cell death), alone or in combination with 10 *μ*M of Boc-D-FMK. (**c**) Caspase-3 activity in Ctrl and RIPK1-depleted L929sA cells measured by DEVD-AMC assay after 24 h of treatment with 0.5 *μ*g/ml of BFAA as well as after 2–4 h of treatment with 30 ng/ml of hTNF (Ctrl cells were stimulated for 4 h and RIPK1-depleted cells for 2 h to induce a comparable amount of cell death). (**d**) RIPK1-depleted (Ctrl/miRIPK1) and RIPK1/RIPK3-depleted (miRIPK1/shRIPK3) L929sA cells were treated for 24 h with 0.5 *μ*g/ml of BFA alone or in combination with 10 *μ*M of Boc-D-FMK, and the cell viability was evaluated by flow cytometry after Sytox Red staining. Cells were also treated with 30 ng/ml of hTNF (2 h). (**e**) Lysates from Ctrl and RIPK1- and RIPK1/RIPK3-depleted L929sA cells probed for RIPK1 and RIPK3 to validate the efficiency of the knockdowns. Actin was used as a loading control. PARP, poly-ADP ribose polymerase

To further characterize the role of RIPK1 during ER stress-induced death, we stably repressed RIPK1 expression in L929sA cells. Surprisingly, and in contrast to the Nec-1 effect, we found that RIPK1 knockdown did not protect the cells from brefeldin A-mediated death (Figure 2a). In the context of TNF signaling, we previously reported that, contrary to Nec-1, RIPK1 repression does not protect L929sA cells from TNF cytotoxicity but instead sensitized them by allowing a switch from necroptotic to apoptotic death.^19, 20^ We therefore wondered whether a similar mechanism was occurring upon ER stress induction. To test this hypothesis, we monitored caspase activation by immunoblot and DEVDase assays in lysates of control and RIPK1-depleted L929sA cells stimulated with brefeldin A, or TNF used as a control. We found that RIPK1 knockdown resulted in the induction of caspase activation both following TNF and brefeldin A stimulation (Figures 2b and c). Interestingly, although Boc-D-FMK was very efficient in inhibiting caspase activation (Figure 2b), it did not protect RIPK1-depleted cells from brefeldin A treatment and only had a limited protective effect following TNF stimulation (Figure 2a). Because a switch back to RIPK3-dependent necroptosis was reported in RIPK1-depleted L929sA cells stimulated with TNF in the presence of caspase-8 repression,^19^ we next investigated the effect of additional RIPK3 depletion on the death of RIPK1-repressing cells stimulated with brefeldin A and Boc-D-FMK. As shown in Figures 2d and e, additional RIPK3 depletion did not protect RIPK1-depleted L929sA cells from brefeldin A used alone but greatly rescued them under caspase-inhibited conditions. As expected, RIPK3 knockdown also provided further protection to the RIPK1-depleted L929sA cells stimulated with TNF and Boc-D-FMK (Figure 2d). Taken together, these results highlight the high similarities in the death pathways activated by TNF and ER stress inducers in L929sA cells.

### MLKL serves as an effector of ER stress-induced necroptosis

**Figure 3:**
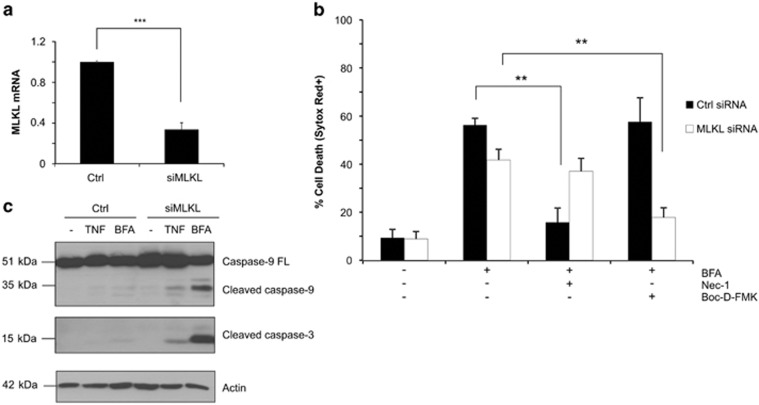
Role of MLKL in ER stress-induced necroptosis. (**a**) Levels of MLKL mRNA after short interfering RNA (siRNA)-mediated knockdown. (**b**) L929sA cells were treated for 24 h with 0.5 *μ*g/ml of brefeldin A (BFA) alone or in combination with 20 *μ*M of Nec-1 or 10 *μ*M of Boc-D-FMK, and viability was evaluated by flow cytometry following Sytox Red staining. (**c**) L929sA cells transfected with control (Ctrl) and MLKL siRNAs were lysed after treatment for 24 h with 0.5 *μ*g/ml of BFA or with 30 ng/ml of human TNF (hTNF) (4 h) and expression of apoptotic markers was analyzed by western blot

MLKL recently emerged as a key molecule mediating necroptosis downstream of RIPK3.^22, 23, 24, 25^ To test whether MLKL has a role in necroptosis triggered by ER stress inducers in L929sA cells, we transfected the cells with MLKL or control siRNA (Figure 3a) and monitored cell viability upon BFA treatment. Interestingly, MLKL repression also resulted in a switch from necroptosis to apoptosis, which was observed by the loss of protective effect of Nec-1 and the gain of protection offered by Boc-D-FMK (Figure 3b). Therefore, contrary to RIPK1, MLKL repression did not allow a switch back to RIPK3-dependent necroptosis in caspase-inhibited conditions. The switch to apoptosis was confirmed by the analysis of caspase-9 and -3 processing by western blot (Figure 3c). As reported previously,^20^ we observed that MLKL repression also induced a switch to apoptosis when stimulating L929sA cells with TNF. Taken together, these results further support activation of the same necroptotic cascade between TNF stimulation and ER stress induction in these cells.

### TNFR1 mediates ER stress-induced necroptosis

**Figure 4:**
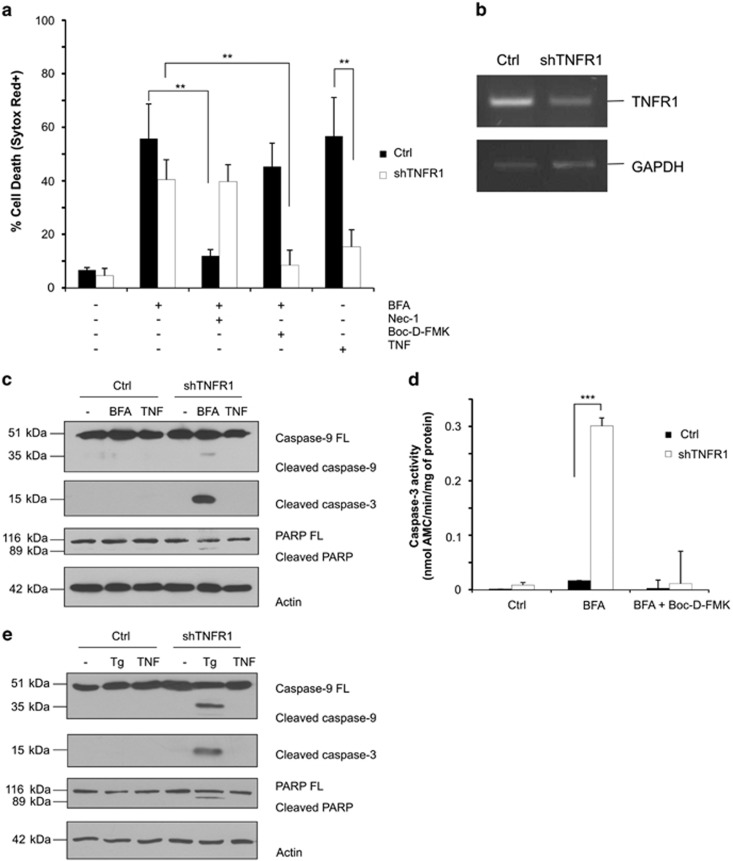
Involvement of TNFR1 in ER stress induced cell death. (**a**) Control (Ctrl) and TNFR1-depleted (shTNFR1) L929sA cells were treated for 24 h with 0.5 *μ*g/ml of brefeldin A (BFA) alone or in combination with 20 *μ*M of Nec-1 or 10 *μ*M of Boc-D-FMK, and viability was evaluated by flow cytometry following Sytox Red staining. Functionality of the knockdown was confirmed after treatment with human TNF (hTNF). (**b**) Knockdown of TNFR1 was validated by reverse transcription-PCR (RT-PCR) with specific primers. Glyceraldehyde 3-phosphate dehydrogenase (GAPDH) was used as an endogenous control. (**c**) Immunoblots of Ctrl and TNFR1-depleted L929sA cells lysates isolated after treatment for 24 h with 0.5 *μ*g/ml of BFA or with 30 ng/ml of hTNF (4 h). (**d**) Caspase-3 activity in Ctrl and TNFR1-depleted L929sA cells measured by DEVD-AMC assay after 24 h of treatment with 0.5 *μ*g/ml of BFA alone or in combination with 10 *μ*M of Boc-D-FMK. (**e**) Immunoblots of Ctrl and TNFR1-depleted L929sA cell lysates isolated after treatment for 24 h with 2.5 *μ*M of thapsigargin (Tg) or with 30 ng/ml of hTNF (4 h). PARP, poly-ADP ribose polymerase

Owing to the strong similarities in the killing of L929sA cells by TNF and ER stress inducers, we wondered whether the death induced by ER stress in these cells would not actually be mediated by TNF itself. Previous studies have indeed reported NF-κB-mediated autocrine production of TNF upon ER stress induction in certain cells.^26^ We therefore first evaluated this possibly by testing the effect of TNFR1 repression on ER stress-induced death. Although not complete, the extent of TNFR1 knockdown that we obtained was sufficient to protect the cells from TNF-induced necroptosis, but had no significant protective effect on brefeldin A cytotoxicity (Figures 4a and b). Nevertheless, the finding that Nec-1 had lost its protective effect in these cells raised the possibility of a switch to another, RIPK1 kinase-independent, cell death modality (Figure 4a). This assumption was confirmed when analyzing caspase activation by immunoblot and DEVDase assays. We observed that TNFR1 repression induced a switch from RIPK1 kinase activity-dependent necroptosis to RIPK1 kinase-independent apoptosis following ER stress induction by brefeldin A or thapsigargin treatment (Figures 4c–e). Importantly, the apoptotic cell death was fully prevented when costimulating the TNFR1-repressing cells with the caspase inhibitor Boc-D-FMK (Figure 3a). These findings therefore demonstrate that TNFR1 is the upstream mediator of necroptosis in L929sA cells undergoing unresolved ER stress, and illustrate the ability of these cells to still switch to apoptosis when TNFR1 signaling is inhibited at the level of the receptor itself.

### TNFR1 mediates ER stress-induced necroptosis independently of ligand binding

**Figure 5:**
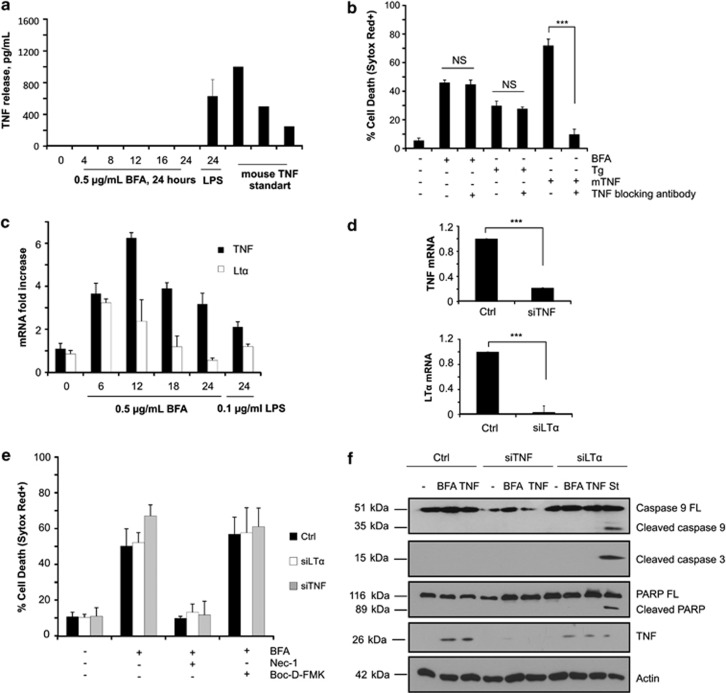
Role of TNFR1 ligands in ER stress-induced necroptosis. (**a**) The release of TNF in the medium after treatment with 0.5 *μ*g/ml of brefeldin A (BFA) for indicated time points was measured by enzyme-linked immunosorbent assay (ELISA). As a control, L929sA cells were also treated with 0.01 *μ*g/ml of lipopolysaccharide (LPS) for 24 h. Mouse TNF standard was used for the standard curve. (**b**) L929sA cells were treated with 20 ng/ml of mTNF (12 h), 0.5 *μ*g/ml of BFA (24 h) or 2.5 *μ*M of thapsigargin (Tg) (24 h) in the presence or absence of 12 ng/ml of TNF-blocking antibody. Viability was evaluated by flow cytometry following Sytox Red staining. (**c**) The induction of TNF and LT*α* mRNAs following treatment with 0.5 *μ*g/ml of BFA for the indicated period of time was evaluated by quantitative PCR (Q-PCR) analysis. (**d**) Levels of TNF and LT*α* mRNA after short interfering RNA (siRNA)-mediated knockdown. (**e**) Control (Ctrl), TNF-depleted (siTNF) and LT*α*-depleted (siLT*α*) L929sA cells were stimulated for 24 h with 0.5 *μ*g/ml of BFA in the absence or presence of 20 *μ*M of Nec-1 or 10 *μ*M of Boc-D-FMK, and cell viability was evaluated by flow cytometry following Sytox Red staining. (**f**) Immunoblots of Ctrl, TNF-depleted and LT*α*-depleted L929sA cell lysates isolated after treatment for 24 h with 0.5 *μ*g/ml of BFA or 4 h with 30 ng/ml of human TNF (hTNF). Cells treated with 10 nM of staurosporine were used as Ctrl for apoptosis induction. NS, not significant; PARP, poly-ADP ribose polymerase

Having established that ER stress kills L929 cells by triggering TNFR1-mediated necroptosis, we next examined whether the killing was occurring as a result of autocrine TNF production. To do so, we first analyzed by ELISA the ability of L929sA cells to secrete soluble TNF following ER stress induction. We found that stimulation with brefeldin A over a period of 24 h did not induce detectable levels of soluble TNF in the cell medium (Figure 5a). By contrast, LPS stimulation, a known inducer of TNF secretion, leads to detectable amounts of TNF in the medium (Figure 5a). To further exclude a role of extracellular TNF, we next incubated the cells with a TNF-blocking antibody before ER stress induction. As shown in Figure 5b, the addition of the TNF-blocking antibody did not affect brefeldin A or thapsigargin cytotoxicity, but efficiently blocked cell death induced by exogenous TNF. Because export of secreted proteins is most probably greatly affected under severe ER stress conditions, we hypothesized that activation of TNFR1 by its ligands could alternatively occur in intracellular compartments. Apart from TNF, lymphotoxin *α* (LT*α*) was recently reported to be as potent as TNF in mediating apoptosis, necroptosis and inflammatory signals.^27^ We therefore analyzed the transcriptional upregulation of the genes encoding these two TNFR1 ligands by Q-PCR analysis. As shown in Figure 5c, brefeldin A treatment resulted in the upregulation of both TNF and LT*α* mRNA transcripts, and therefore still supporting a potential role of their encoded proteins in ER stress-induced death. We then analyzed the effect of repressing TNF and LT*α* on the killing potential of brefeldin A. The efficacy of TNF and LT*α* knockdown was confirmed at the mRNA level by Q-PCR (Figure 5d) and at the protein level by western blotting for TNF (Figure 5f). We found that TNF and LT*α* repression had no effect on brefeldin A cytotoxicity when compared with nonspecific siRNA treatment (Figure 5e). Importantly, we confirmed that the lack of protective effect was not due to a switch to RIPK1 kinase-independent apoptosis, as observed upon TNFR1 repression. Indeed, contrary to TNFR1-depleted cells (Figure 4), brefeldin A treatment did not induce caspase activation in the TNF- and LT*α*-depleted cells (Figure 5f). Accordingly, these cells were still protected by Nec-1, while Boc-D-FMK had no effect on their viability (Figure 5e). These results clearly demonstrate that ER stress induces TNFR1-mediated necroptosis in L929sA cells independently of ligand binding.

### Role of the JNK pathway in ER stress-mediated cell death

**Figure 6:**
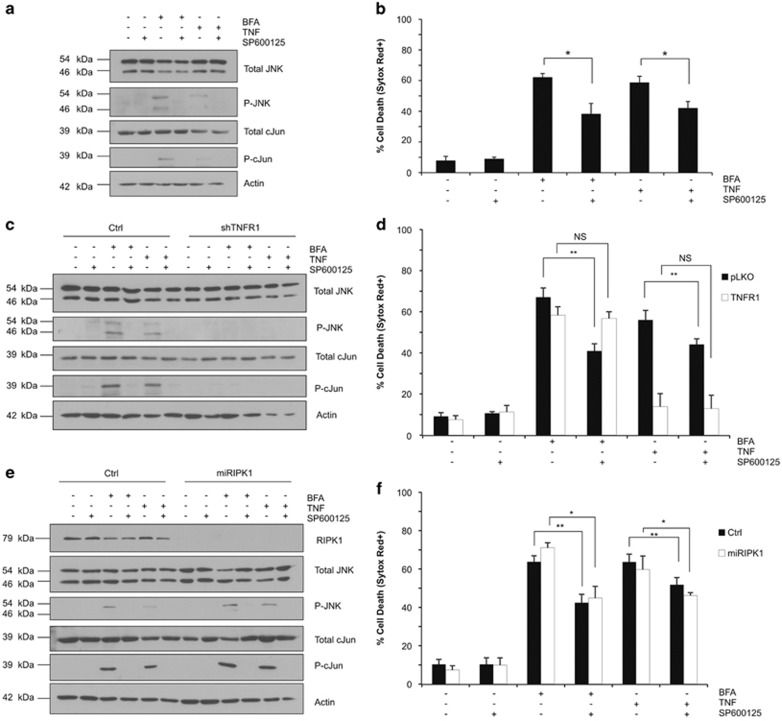
Role of JNK inhibition in ER stress-induced cell death. (**a** and **b**) L929sA cells and (**c** and **d**) control (Ctrl) and shTNFR1 L929sA cells were stimulated for 24 h with 0.5 *μ*g/ml of brefeldin A (BFA) or 4 h with 30 ng/ml of human TNF (hTNF) in the absence or presence of 20 *μ*M of SP600125. (**a** and **c**) Cell lysates were immunoblotted as indicated and (**b** and **d**) cell viability was evaluated by flow cytometry following Sytox Red staining. (**e** and **f**) Control (Ctrl) and miRIPK1 L929sA cells stimulated for 24 h with 0.5 *μ*g/ml of brefeldin A or with 30 ng/ml of hTNF (Ctrl cells were stimulated for 4 h and RIPK1-depleted cells for 2 h to induce a comparable amount of cell death) in the absence or presence of 20 *μ*M of SP600125. (**e**) Cells lysates were immunoblotled as indicated and (**f**) cell viability was evaluated by flow cytometry following Sytox Red staining

It has previously been suggested that ER stress could kill by activating a TRAF2/JNK pathway,^2^ and that RIPK1 was required for JNK activation by interacting with TNFR1.^28^ Knowing the contribution of JNK to both apoptotic^29^ and caspase-independent mode of cell death,^30^ we therefore evaluated the role of the JNK pathway in our cellular model. To do so, we applied SP600125, an inhibitor of JNK-1, -2 and -3,^31^ in combination with BFA or TNF treatment in L929sA cells and evaluated its effect on the viability of the cells. We observed that SP600125 successfully inhibited phosphorylation of JNK, as well as its downstream target c-Jun, in response to both BFA and TNF (Figure 6a), and provided partial protection to necroptosis induced by both triggers (Figure 6b). Importantly, we observed that JNK activation was a result of TNFR1 signaling following BFA and TNF treatments. Indeed, TNFR1 repression by shRNA prevented BFA- and TNF-mediated phosphorylation of JNK and c-Jun (Figure 6c), and resulted in the loss of the protective effect of SP600125 (Figure 6d). Nevertheless, we found that RIPK1 was not required for JNK activation (Figure 6e), and consequently that SP600125 also provided partial protection to apoptosis induced by TNF and BFA in the RIPK1-repressing cells (Figure 6f). Taken together, these results indicate that ER stress induction in L929sA cells activates ligand-independent TNFR1 signaling that mediates both RIPK1/RIPK3/MLKL-dependent necroptosis and RIPK1-independent JNK-dependent death.

## Discussion

Cell death is a crucial process for multicellular organisms, as it ensures proper morphogenesis, establishment of the immune system, elimination of damaged cells and maintenance of homeostasis. As a consequence, cell death needs to be tightly regulated because inappropriate cell death responses inexorably lead to the development of pathologies. In humans, these include neurodegenerative disorders, autoimmune diseases, diabetes and cancers. Apoptosis, a process relying on the activation of the caspase cascade, has long been considered the only form of regulated cell death, but existence of additional forms of controlled cell death is now well established.^32, 33^ These can be triggered independently of apoptosis induction or as back-up safety mechanisms *in situations* where the apoptotic machinery does not operate properly, such as a result of genetic mutations or chemical/microbial inhibition.

Necroptosis, or programmed/regulated necrosis, is a non-apoptotic, caspase-independent, inflammatory type of cell death that relies on the enzymatic activity of RIPK1/3 and on the pseudokinase MLKL.^14, 15^ Necroptosis has attracted a lot of attention lately because of its demonstrated role as alternative cell death modality during infection,^34^ as well as for its contribution to the pathogenesis of several human diseases, such as ischemic brain injury, myocardial infarction and stroke, renal ischemia–reperfusion injury, pancreatitis and inflammatory bowel diseases (for a review see Linkermann and Green^14^and Vanlangenakker *et al.*^35^). Interestingly, conditions such as infection and hypoxia–ischemia are physiological ER stress inducers, and ER stress-induced cellular dysfunction and death have been associated with diseases that are known to be mediated, at least in part, by necroptosis.^6, 7^ However, whether ER stress could directly trigger necroptosis had remained an open question. Indeed, studies in the ER stress field have so far mainly focused on the the ability of ER stress to kill by activating the intrinsic apoptotic pathway.^4^ In this study, we provide clear evidence that unresolved ER stress, induced by three different compounds, can also result in necroptosis induction, thus highlighting a potential molecular link between ER stress, necroptosis and the establishment of those diseases. In addition, the fact that ER stress can trigger an inflammatory type of cell death, which contrasts with apoptosis that is in many conditions immunosilent, opens doors for future studies on the role of ER stress in inflammation-driven pathologies.

In our cellular system, the L929sA cell line, we found that ER stress-induced necroptosis was mediated by TNFR1, which is in line with earlier studies implicating TNFR1 signaling in the cellular response to ER stress.^26, 28^ As previously reported following TNF stimulation,^19^ we observed that RIPK1 kinase inhibition by Nec-1, or Nec-1s, protected L929sA cells from ER stress cytotoxicity while RIPK1 repression induced a switch to apoptosis. The plasticity in the cell death modality used was further highlighted by the switch back to RIPK1-independent necroptosis when caspases were additionally inhibited. Indeed, RIPK1-depleted L929sA cells could only be rescued from ER stress-induced death by the combination of caspase inhibition and RIPK3 repression. In contrast, MLKL repression induced a switch to apoptosis that could be inhibited by caspase inhibition, highlighting the more downstream role of MLKL in the necroptotic cascade. Importantly, the ability of the cells to adapt to an alternative cell death route was not only observed upon repression of a downstream executioner but also when knocking down the receptor itself. Indeed, a switch from necroptosis to apoptosis was also detected upon TNFR1 repression. Taken together, the results highlight the difficulty to block cell death under ER stress conditions owing to the ability of the cells to circumvent blockade of one specific pathway by activating alternative death routes. These findings are of major importance for the success of therapeutic strategies aimed at inhibiting ER stress-mediated death. In this context, our results show that Nec-1 is a good candidate, as the allosteric inhibition of RIPK1 kinase activity does not allow a switch to another death mode. Moreover, these results also highlight the importance of proper cell death typing when evaluating the contribution of certain proteins in the death induced by ER stress.

Surprisingly, we found, using blocking antibody and RNAi approaches, that signaling by TNFR1 during ER stress was occurring independently of ligand binding. These results are in contrast with those from Hu *et al.*^26^ who suggested a model of ER stress-induced TNFR1 activation resulting from IRE1-mediated NF-*κ*B-dependent autocrine production of TNF.^26^ Nevertheless, our results support the study of Yang *et al.*,^28^ which suggested ligand-independent activation of TNFR1 at the ER membrane during ER stress.^28^ Interestingly, ligand-independent signaling by TNFR1 at the ER was also recently reported in the case of TNF receptor-associated periodic syndrome (TRAPS), an autosomal-dominant autoinflammatory disease associated with heterozygous mutations in TNFR1.^36^ In this study, the authors showed that TRAPS-associated mutant TNFR1 molecules are retained in the ER and unable to bind TNF but still capable of signaling to NF-κB and to cell death. Of note, in our study, it is unknown whether the ER stress-induced ligand-independent TNFR1 signaling originates at the plasma membrane or intracellularly. However, knowing that severe ER stress conditions alter export of membrane proteins, it is reasonable to speculate that it also originates from intracellular compartments, and potentially from the ER. Of note, ER stress-induced ligand-independent DR5-mediated apoptosis has recently been reported in other cell types.^37^ This demonstrates the ability of ER stress to trigger ligand-independent activation of death receptors, whose identity may vary depending on the cell type.

Finally, we observed that activation of TNFR1 by ER stress or TNF stimulation in L929sA cells resulted in JNK activation, which partially contributed to necroptosis induced by these triggers. In contrast to the results of Yang *et al.*,^28^ we found that RIPK1 repression had no impact on JNK activation, and that JNK inhibition still provided protection to apoptosis resulting from RIPK1 repression in these cells. The fact that JNK inhibition provided protection to both TNFR1-induced necroptosis and apoptosis is intriguing, and suggest a rather aspecific role of JNK in cell death induction in these cells.

In conclusion, our study reveals the ability of ER stress to trigger RIPK1 kinase-dependent necroptosis, which opens new doors for future work on the implication of ER stress in pathologies resulting from the inappropriate induction of this inflammatory type of cell death.

## Materials and Methods

### Cell culture and reagents

L929sA cells were cultured in Dulbecco's modified Eagle's medium (Sigma-Aldrich, St. Louis, MO, USA; D6429) with 10% (v/v) fetal bovine serum, 1% (v/v) penicillin–streptomycin (Sigma-Aldrich; P0781) and 2 mM l-glutamine (Sigma-Aldrich; G7513). HEK293T cells used for the viral production were grown in the same media but without the l-glutamine supplement. Cells were cultured in a humidified incubator at 37 °C with a 5% CO_2_ concentration. The reagents used for the treatments were as follows: human TNF (Immunotools, Friesoythe, Germany; no. 11343017), staurosporine (Sigma-Aldrich; no. S6942), brefeldin A (Sigma-Aldrich; no. B7651), tunicamycin (Sigma-Aldrich; no. T7765), thapsigargin (Sigma-Aldrich; no. T9033), Boc-D-FMK (Cambridge Bioscience, Cambridge, UK; no. 1120-20C), Nec-1 (Santa Cruz Biotechnology, Dallas, TX, USA; no. CAS 4311-88-0), RIP1 inhibitor II, 7-Cl-O-Nec-1 (Nec-1s) (Merck, Darmstadt, Germany; no. 504297) and SP600125 (Sigma-Aldrich; no. S5567). Recombinant mouse TNF is produced and purified to at least 99% homogeneity in our laboratory. The antibodies used in the study were as follows: PARP (CST, Danvers, MA, USA; no. 9542), caspase-9 (CST; no. 9508), cleaved caspase-3 (CST; no. 9665), actin (Sigma-Aldrich; no. A2066), RIPK1 (BD Transduction Laboratories, Franklin Lakes, NJ, USA; no. 610459), RIPK3 (Sigma-Aldrich; no. R4277), TNF (Santa Cruz Biotechnology; no. sc-52746), c-Jun (Santa Cruz Biotechnology; no. sc-1694), phospho-c-Jun (Santa Cruz Biotechnology; no. sc-822), SAPK/JNK (CST; no. 9252) and phospho-SAPK/JNK (CST; no. 9251). All the secondary antibodies were purchased from The Jackson Laboratory (Bar Harbor, ME, USA) and the signal was visualized using Western Lightning ECL substrates (Perkin-Elmer, Waltham, MA, USA). The TNF-blocking antibody comes from Bioceros BV (Utrecht, Netherland; no. XT-22). The mouse TNF ELISA Kit was purchased from eBioscience (San Diego, CA, USA; no. 88-7324-22) and used according to the manufacturer's protocol.

### Gene repression

Control, miRIPK1 and miRIPK3 L929sA cells were generated as described previously.^38^ pLKO lentiviral control and shRNA vector against mouse TNFR1 (TRCN0000066103) were obtained from Sigma-Aldrich. Lentivirus production was obtained by co-transfecting the lentiviral plasmids with the second-generation lentivirus packaging system (Addgene, Cambridge, MA, USA; pMD2.G cat. no. 12259, psPAX2 cat. no.12260, pRSV-Rev cat. no. 12253) using JET PEI transfection reagent (Polyplus Transfection, Illkirch, France; cat. no. 101-01N) into HEK293T cells. Virus-containing supernatant was harvested and filtered through 0.22 *μ*m filter. Transduction was performed in the presence of 5 *μ*g/ml of polybrene (Merck Millipore, Darmstadt, Germany; TR-1003-G). Control and shTNFR1 cells were selected for 72 h in 5 *μ*g/ml of puromycin. TNF (L-042302-00-0005), LT*α* (L-043958-00-0005) and nonspecific (D-001810-01-20) siRNAs were purchased from Dharmacon (Thermo Fisher Scientific, Waltham, MA, USA). siRNA transfection was performed using INTERFERin (Polyplus Transfection; no. 409-50) according to the manufacturer's protocol.

### RNA extraction, RT-PCR and Q-PCR

RNA was extracted using the TRIzol reagent (Sigma-Aldrich; no. T9424), precipitated with ethanol and reverse transcribed using the superscript III following the manufacturer's protocol (Invitrogen, Waltham, MA, USA). The obtained cDNA was used as template for the PCR reactions using the following primers: GAPDH (forward primer: 5′-ACCACAGTCCATGCCATC-3′ reverse primer: 5′-TCCACCACCCTGTTGCTG-3′) and TNFR1 (forward primer: 5′-CAGTCTGCAGGGAGTGTGAA-3′ reverse primer: 5′-CACGCACTGGAAGTGTGTCT-3′) (Integrated DNA Technologies, Coralville, IA, USA).

Q-PCR was performed using Brilliant III Ultra-Fast Q-PCR Master Mix (Agilent, Santa Clara, CA, USA) according to the manufacturer's protocol. Relative gene expression was evaluated by ΔΔCT method and GAPDH was used as the housekeeping gene to normalize gene expression. Probes were purchased from Integrated DNA Technologies and were as follows: GAPDH (forward primer: 5′-GCCTTCCGTGTTCCTACC-3′ reverse primer: 5′-CCTCAGTGTAGCCCAAGATG-3′), LT*α* (forward primer: 5′-TCTCCAGAGCAGTGAGTTCT-3′ reverse primer: 5′-CTCAGAAGCACTTGACCCAT-3′), TNF (forward primer: 5′-TCTTTGAGATCCATGCCGTTG-3′ reverse primer: 5′-AGACCCTCACACTCAGATCA-3′) and MLKL (forward primer: 5′-TCTCTCTGCTTTAGTGCTCTTTG-3′ reverse primer: 5′-CAGCTCCAGTTTCCTCGTAG-3′).

### DEVDase assay

Following stimulation, the cells were trypsinized, pelleted, washed once with PBS and resuspended in 50 *μ*l of ice-cold PBS. Samples were then snap-frozen in duplicate and kept at −80 °C until further processing. On the day of the assay, the samples were diluted in the assay buffer (100 mM HEPES, 10% sucrose and 0.1% CHAPS) in the presence of a final concentration of 50 *μ*M of Ac-Asp-Glu-Val-Asp-*α*-(4-methyl-coumaryl-7-amide) (Peptide Institute Inc., Osaka, Japan; cat. no. 3171-v), 0.0001% NP-40 and 5 *μ*M of DTT. The samples were then read at 355 nm excitation and 460 nm emission on a preheated plate reader (at 37 °C) for 30 cycles with 1 min intervals.

### Cell death analysis

Following stimulation, the cells were trypsinized, pelleted, resuspended in PBS containing 5 nM of Sytox Red (Invitrogen; no. S34859) and incubated for 15 min on ice. The cells were then analyzed for Sytox Red positivity using the BD FACs Canto Flow Cytometer (BD Bioscience, Franklin Lakes, NJ, USA). The percentage of cell death is calculated as follows: % cell death=(no. of Sytox Red^+^/(no. of Sytox Red^+^+no. of Sytox Red^−^)) × 100.

### Statistical analysis

Experiments were repeated independently at least three times. Error bars represent the standard deviation (S.D.) of replicates. Statistical analyses were performed using unpaired Student's *t*-tests using the GraphPad Software (San Diego, CA, USA) (*P<0.05, **P<0.01 and ***P<0.001).

## Abbreviations

BCL-2: B-cell lymphoma 2
CHOP: C/EBP-homologous protein
ER: endoplasmic reticulum
IRE1: inositol-requiring enzyme-1
JNK: c-Jun N-terminal kinase
LT*α*: lymphotoxin *α*
MOMP: mitochondrial outer membrane permeabilization
MLKL: mixed lineage kinase domain-like protein
PERK: protein kinase RNA-like ER kinase
RIPK: receptor-interacting protein kinase
TNF: tumor necrosis factor
UPR: unfolded protein response
