# Supplementary material for: Endoplasmic reticulum stress induces ligand-independent TNFR1-mediated necroptosis in L929 cells
Source: Cell Death Dis. 2015 Jan 8;6(1):e1587–. doi: 10.1038/cddis.2014.548 (PMC4669746; doi:10.1038/cddis.2014.548)
Supplement: Supplementary Figures [file cddis2014548x1.doc]

Supplemental information.


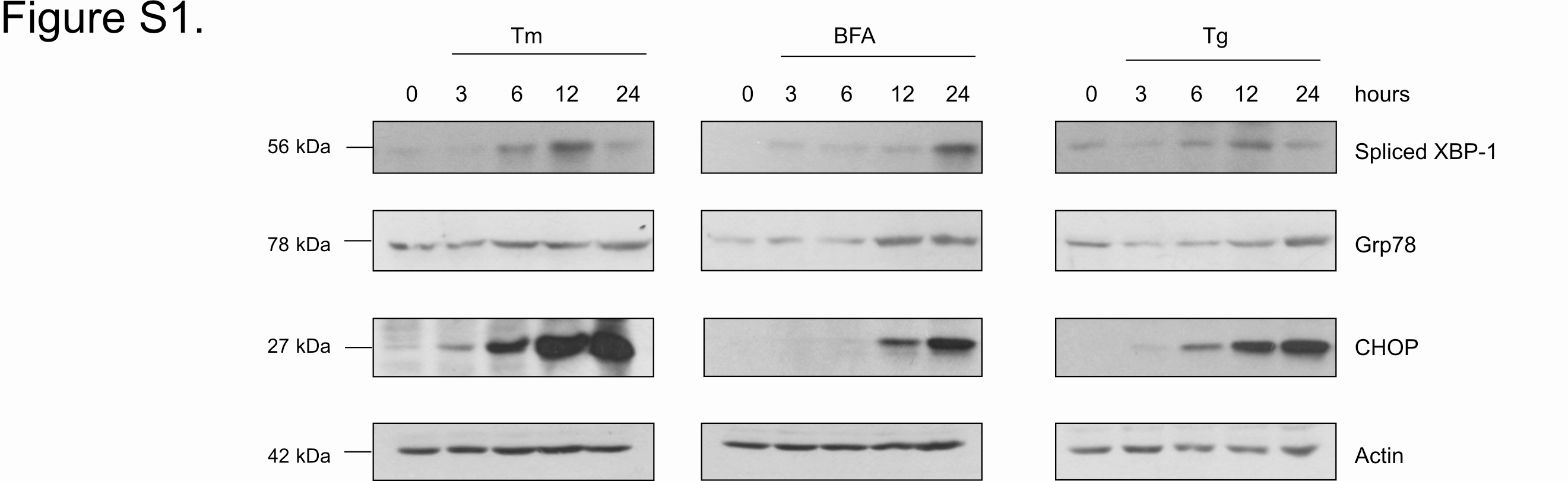


**Fig. S1.** **Tunicamicin, brefeldin A and thapsigargin induce ER stress in L929sA cells.** L929sA cells were treated with 2.5 μg/mL of tunicamycin, 0.5 μM of brefeldin A, or 2.5 μM of thapsigargin for indicated times. Lysates were harvested and immunoblotted with antibodies against spliced XBP-1, Grp78 or CHOP. Actin was used as a loading control.


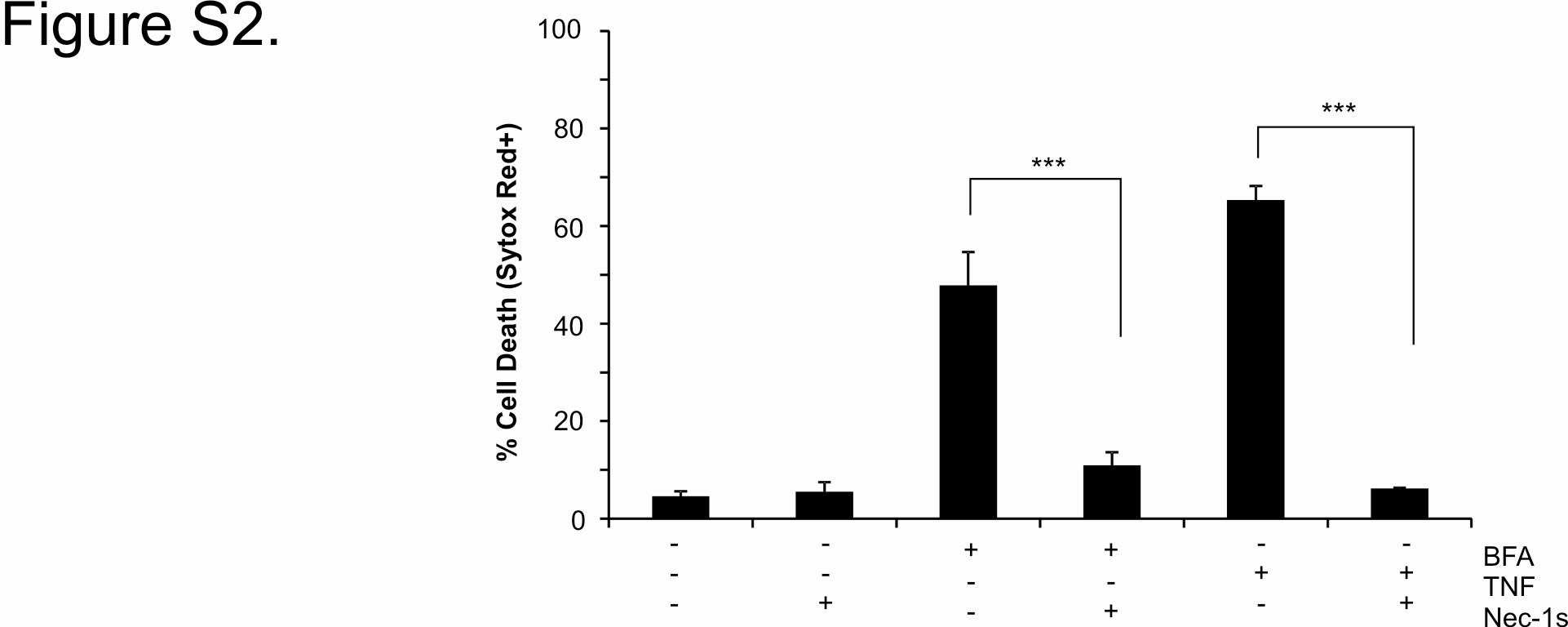


**Fig. S2.** **ER stress-induced death of L929sA cells is inhibited by Nec-1s.**

Cell death was evaluated by flow cytometry after Cytox Red staining of L929sA stimulated with 0.5 μg/mL of brefeldin A (24h) and 30 ng/mL of hTNF (4h) in the absence and presence of 5 μM of RIP1 Inhibitor II (Nec-1s).
